# Hello, kitty: could cat allergy be a form of intoxication?

**DOI:** 10.1590/1678-9199-JVATITD-2020-0051

**Published:** 2020-12-14

**Authors:** Rodrigo Ligabue-Braun

**Affiliations:** 1 Department of Pharmacosciences, Federal University of Health Sciences of Porto Alegre (UFCSPA), Porto Alegre, RS, Brazil.

**Keywords:** Allergy, Cat, Domestication, Fel d 1, Loris, Secretoglobin

## Abstract

**Background:**

The relationship between slow loris (*Nycticebus* spp.) venom (BGE protein) and the major cat allergen (Fel d 1) from domestic cat (*Felis catus*) is known for about two decades. Along this time, evidence was accumulated regarding convergences between them, including their almost identical mode of action.

**Methods:**

Large-scale database mining for Fel d 1 and BGE proteins in Felidae and *Nycticebus* spp., alignment, phylogeny proposition and molecular modelling, associated with directed literature review were assessed.

**Results:**

Fel d 1 sequences for 28 non-domestic felids were identified, along with two additional loris BGE protein sequences. Dimer interfaces are less conserved among sequences, and the chain 1 shows more sequence similarity than chain 2. Post-translational modification similarities are highly probable.

**Conclusions:**

Fel d 1 functions beyond allergy are discussed, considering the great conservation of felid orthologs of this protein. Reasons for toxicity being found only in domestic cats are proposed in the context of domestication. The combination of the literature review, genome-derived sequence data, and comparisons with the venomous primate slow loris may point to domestic cats as potentially poisonous mammals.

## Background

Toxicity caused by mammals is a relatively obscure subject. Mammals are known to be venomous at least since the 1800s [1]. This fact, however, remained majorly underappreciated until very recently [2-5]. Still more intriguing is the fact that almost no poisonous mammal has been described. Records of mammalian poisons are restricted to intoxication by consumption of sea mammal liver [6], and sequestration of exogenous toxins in modified hair in hedgehogs and in the African crested rat [7-9]. As defined by Brodie [10] in regards to animal toxins, poisons are passively encountered and do not have any special mechanism of delivery into the body of another organism, while venoms are molecular blends housed and produced in specialized structures that are associated with a delivery device.

In this paper, I present the hypothesis that domestic cats (*Felis catus*) can be considered poisonous mammals. This proposition involves Fel d 1, the major cat allergen, which has functions underappreciated outside the allergy context. The proposition of a mammalian poison produced by cats has its genesis in the slow loris (*Nycticebus* spp.), a venomous mammal with a very elaborate envenomation apparatus [4]. Different species of slow loris synthesize the BGE protein in the brachial gland (hence, brachial gland exudate or BGE protein), which is licked, and mixed with saliva, filling up specialized incisor teeth that work as needles [4, 11]. When bitten by the animal in such “loaded” state, humans (and other animals, including loris conspecifics) have varied physiological responses, from nothing to tissue decay, anaphylactic shock, and death [3,4]. The BGE protein has been recently shown to closely resemble Fel d 1, the major cat allergen [12,13]. This connection between venom and allergen led to an inspection of Fel d 1 in a broader, physiological context, since no pinpointed function has been ascribed to this protein [14].

Discovered in 1973 [15], Fel d 1 is an oligomeric protein composed by two heterodimers, being described as a dimer of dimers. The all-helical monomers from chain 1 and chain 2 (NCBI gene ID 677879 and 677877, respectively) associate in heterodimers that assume the U-fold of the secretoglobin family, which is highly similar to the traditional globin fold [16,17]. The name of this family derives from the fact that the proteins are present at high levels in mammalian secretions from pulmonary, uterine, prostatic, lacrimal, and salivary origin (and probably others) [18]. The secretoglobin fold forms a hydrophobic binding cavity, shown in other proteins in the family to bind steroid hormones, retinoids, eicosanoids, and polychlorinated biphenyl metabolites [19]. Chain 2 has an Asn-glycosylation site, and multiple Fel d 1 glycoforms have been shown to exist [20,21].

Fel d 1 is part of a set of allergens from domestic cats (named Fel d 1 to Fel d 8), being the main responsible for allergic responses in humans. Recent sensitivity comparisons estimated Fel d 1 as causing up to 95% of the observed effects of all cat allergens. Cats, present in up to half of all households in the world, are the second major cause of indoor allergies, being surpassed only by mites [22,23]. It is estimated that 10-15% of all adults are sensitized to Fel d 1, presenting symptoms that range from mild rhinoconjunctivitis to life-threatening respiratory complications [24].

The protein is found in different cat anatomical sites, including skin, fur, mammary, salivary, sebaceous and anal glands [25-28]. The highest levels are found in anal glands, followed by fur and saliva [26,28]. Fel d 1 from different sources may be mixed with the one found in saliva, and deposited on skin and fur, since cats use their highly specialized tongue, equipped with hollow papillae, to wick up saliva [29].

The allergy-causing role has been the main research focus in Fel d 1 studies. This protein, however, has other functions that aid in the comprehension of its physiological role and highlight its similarities to the toxic loris BGE protein.

Considering the similarities between primate BGE and cat Fel d 1, here I present a working hypothesis that cats may employ Fel d 1, the major cat allergen, as a defense mechanism, and as an intra- and interspecific communication tool. The rationale for this proposition, along with supporting evidence and their possible shortcomings, are discussed.

## Methods

To inspect for presence and variability of Fel d 1 in non-domestic felids, here I present the first full-scale database mining focused on this protein. Using the reference sequences for the domestic cat Fel d 1 chain 1 (UniProtKB - P30438) and chain 2 (UniProtKB - P30440), BLAST searches [30,31] were performed against protein, nucleotide, genome, and short reading databases at NCBI [32], and filtered for data pertaining to Felidae (NCBI:txid9681). Sequence alignments were performed with MUSCLE [33], sequence manipulations were performed with AliView [34], phylogenetic analyses were performed with PhyML, under maximum likelihood, following the JTT+G substitution model and branch support estimation by aLRT [35-38]. Tridimensional structure visualization and manipulation were carried out with UCSF Chimera [39]. These Fel d 1 sequence and structure data were combined with directed literature review to elaborate the hypothesis presented in this work.

## Results

**Figure 1.**
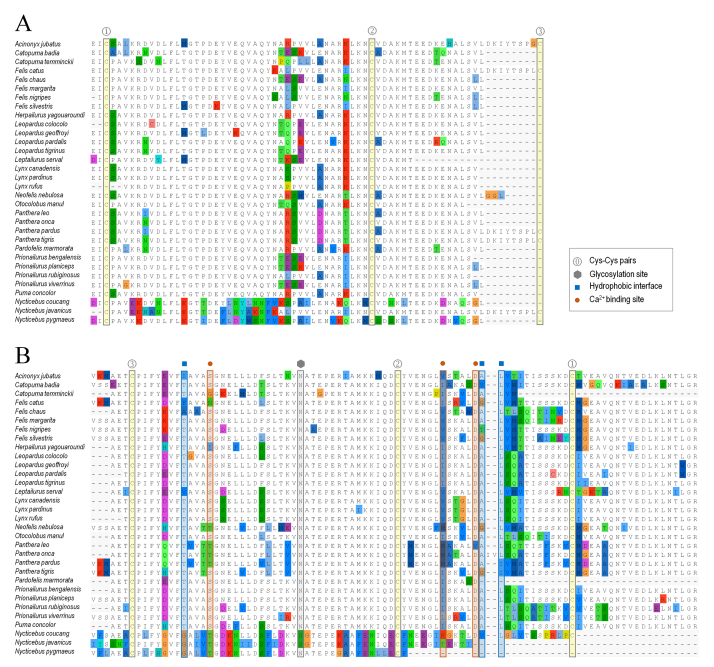
Sequence alignment for felid Fel d 1 and loris BGE protein. **(A)** Chain 1, **(B)** chain 2. Characters are colored highlighting differences from majority rule consensus. Structurally relevant positions are highlighted according to the legend box and include Cys-Cys pairs, interface hydrophobic residues, Ca^2+^ binding residues and a glycosylation site.

**Figure 2.**
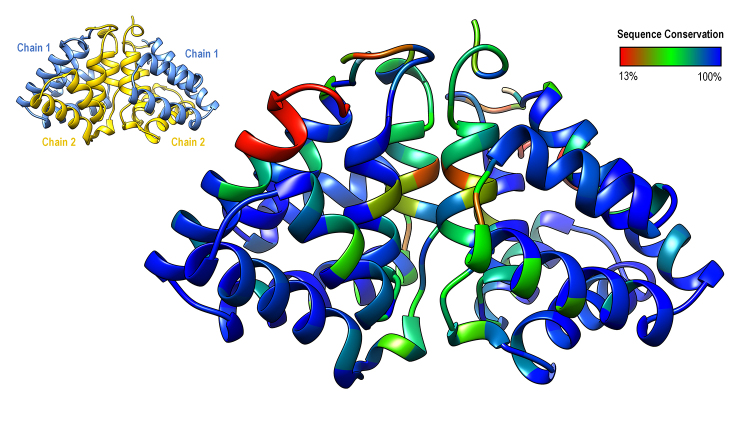
Sequence conservation mapped onto Fel d 1 structure. The information from sequence alignments (Figure 1) was used to locate tridimensionally the positions of greater amino acid conservation. Structure based on PDB ID: 2EJN [17]. A scheme indicating the orientation of each dimer in the tetramer (dimer-of-dimers) is also shown.

Fel d 1 sequences for 28 species were found, covering all Felidae groups [40,41]. Sequence IDs, species and common names are presented in Additional file 1 (species for which there are insufficient or unavailable data are shown in Additional file 2). Here, besides the full sequence of *N. javanicus* BGE protein recently obtained by Scheib et al. [13], two additional sequences, for *N. coucang* and *N. pygmaeus*, were found by database mining. The sequence alignments (Figure 1) reveal the high conservation of felid Fel d 1 and their more distant similarity to sequences for slow loris (*Nycticebus* spp., NCBI: txid9469). The glycosylation site is conserved for all species, with a proposed shift from N- to O-glycosylation in *N. javanicus* [13] being also found for *N. coucang*. One of the disulfide bonds (Cys pair 3) is not conserved in these alignments, due to shorter chain 1 sequences for most of the inspected species. Despite its recurrence, the shortening of sequences at their C-terminus due to genetic sequencing issues cannot be discarded. Calcium ion binding sites [17,21] are more conserved than the interface hydrophobic cluster [13,17]. The Fel d 1 dimer-of-dimers interface is less conserved than the core cavity-bearing dimers, as shown in Figure 2.

**Figure 3.**
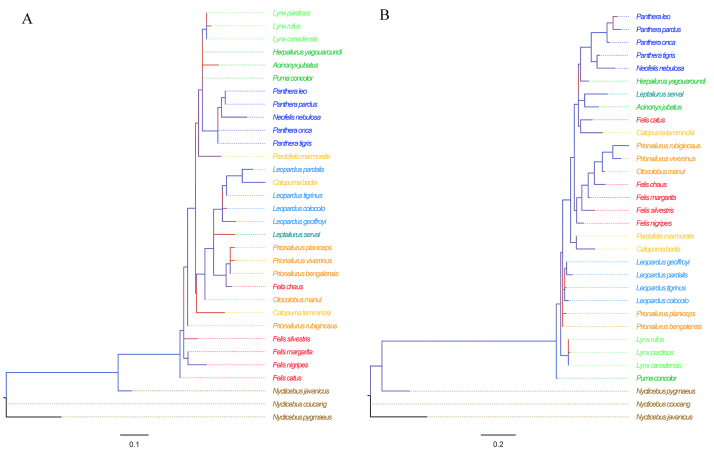
Phylogenetic analyses of felid Fel d 1 and loris BGE protein. **(A)** Chain 1, **(B)** chain 2. Felid species are colored according to their current grouping [41]. Branch support is shown as aLRT gradient.

The lower similarity observed for chain 2, where most of the interface residues are found, in comparison with chain 1 is also supported by phylogenetic analyses on both chains of Fel d 1 (Figure 3). While a closer relationship between slow loris (*Nycticebus* spp.) and domestic cat (*F. catus*) sequences is indicated for chain 1, the same is not observed for chain 2. Such difference can indicate that chain 1 holds most of the toxic activity, that could be retained between lorises and cats, while chain 2, including its interface binding residues, would be less relevant for this specific activity.

## Discussion

The allergenic potential of Fel d 1 is well recognized and has been the theme of multiple reviews [23,24]. In the present study, I highlight the specific connections between allergy and toxins, and their relevance in the context of potential cat toxicity.

Allergies are generally considered as an exaggerated response due to hypersensitivity of the immune system to (usually) innocuous substances in the environment [42]. They are mediated by Immunoglobulin E (IgE), which is allergen-specific and signals to mast cells to release multiple pro-inflammatory molecules once the individual re-encounters an allergen [43,44]. The association of IgE and defense against toxins is acknowledged in recent literature [45,46], but as a minor function, with allergy being its separate, major role. Thus, allergies are generally considered an overblown response that is not expected in most of the population, and their effects would be an evolutionary burden. This view has been challenged by Profet [47], whose proposition is that allergy-propensity is an advantageous trait that protects the individual from environmental toxins. Current developments of this suggestion list multiple mechanisms of allergy-based individual defenses, including barrier enhancement (via keratinocyte and goblet cell hyperplasia with mucus secretion), removal/expulsion of insulting substance (via sneezing, coughing, vomiting, diarrhea, and itch), restriction (via granuloma formation, for instance), and conditioned avoidance against venomous and poisonous species [48]. It is in this theoretical framework that Fel d 1 toxicity is proposed.

Besides resistance to several endo- and ectoparasites [49], there is experimental evidence that allergies/Ig-E mediated responses are involved in enhancement of innate response to arthropod and reptilian venoms. Such resistance (almost like a “vaccination”) was shown in murine models of injection with venoms from either honeybee (*Apis mellifera*), Gila monster (*Heloderma suspectum*), Israeli mole viper (*Atractaspis engaddensis*), or Russell's viper (*Daboia russelii*) [50, 51]. With escalating doses of injected venom, rats and mice were shown to eventually resist to otherwise lethal quantities of toxin.

Allergies and anaphylactic shock are well-established for snake bites [52-54] and arthropod stings [55]. These examples, involving venoms which are actively injected by the inflicting animal, are not directly correlated with Fel d 1-mediated cat allergy. Nevertheless, both snake and arthropods can elicit allergy when externally contacting the human body. Multiple insects and arachnids have been shown to cause allergies that are unrelated to stinging or any form of “active” toxicity (i.e. venom) [56]. Likewise, cutaneous, ocular, and respiratory exposure to venoms from spitting cobra (*Hemachatus hemachatus*) and South American Crotalinae vipers (*Bothrops asper*, *B. atrox*, *B. jararaca*, *B. xanthograma*, *Crotalus durissus terrificus*, *Lachesis muta*) originate allergenic responses [57-61].

It has been argued that allergens constitute a definite set of antigens, specifically those that are homologous to parasite proteins (e.g. from intestinal helminths) [62]. Fel d 1, however, is a secretoglobin, a family of proteins restricted to mammals [63]. In addition, it is disulfide-rich [16], a characteristic found in some respiratory allergens [64], and common in toxins found in animal venoms [65,66]. The disulfide bonding may explain Fel d 1 heat stability [67] and why it is so environmentally persistent. It has been found in dwellings, classrooms, cinemas, hotels, cars, buses, and clothing [68-73]. It has even been detected in the isolated Tristan da Cunha Island twenty years after all cats were removed from its territory [74], and in the Greenland inland ice shelf, were cats are unlikely to have lived [13]. Fel d 1 is found in particle sizes as small as 4.7 µm, making it suitable for airborne transportation [75,76]. Vacuum and steam cleaning were shown to be inefficient in removing the protein from domestic environments [70,77], while the use of high efficiency particulate air (HEPA) filters was able to reduce its levels [78]. Washing cats was shown to temporarily reduce free protein levels [79]. These characteristics make Fel d 1 virtually unavoidable for the affected individuals [67].

Besides its allergy-inducing abilities, Fel d 1 has been shown to have lipid binding properties that may be involved in intra- and interspecific communication [21,27, 80,81]. Fel d 1 has been shown *in silico* and *in vitro* to bind multiple hydrophobic ligands, including androstenone, pregnenolone, progesterone, lauric, oleic, linoleic, and myristic fatty acids [21, 81], in agreement with binding tendencies observed for other secretoglobins [81,82]. Their function, however, is still elusive, with ‘secreto’ having the double meaning of ‘secretory’ and ‘mysterious/secret’ [19]. Previously shown to be likely homologues [83], comparisons of Fel d 1 and mouse salivary ABP (androgen-binding protein) demonstrated extensive similarities between them, pointing to a comparable evolutionary origin and possible functional constraints [14].

The facial and anal sites of Fel d 1 deposition are consistent with pheromone-releasing sites involved in cat intraspecific communication [84], and this co-localization led to the proposal of Fel d 1 as capable of binding pheromones and being involved in intraspecific communication [27]. The similarity between Fel d 1, ABP, and some other pheromone-binding proteins [14], along with the specificity of Fel d 1 to various semiochemicals [81], support its role in intraspecific communication. An additional evidence for this action is that Fel d 1 levels vary if cats are either male or female, neutered or non-neutered, handling-avoidant or sociable. The general trend is to find higher protein levels in non-neutered, handling-avoidant males [28,80]. Deviations of this pattern, in which sociable females had higher levels of Fel d 1 than handling-avoidant females are thought to reflect female cat interactions with humans, which are considered more elaborate than male’s [80].

Besides intraspecific communication, there is growing evidence that Fel d 1 acts on interspecific communication. Rats are able to identify individual cats based on their collars [85], and different experimental conditions were used to show that cat body rubbings elicit defensive behavior in rats [86]. Since Fel d 1 is the major component of cat dander [23], it is reasonable to consider that rodents may be sensitive to this protein. In this context, Fel d 1 would act as a kairomone [86], a chemical sign (originally a pheromone) in the predator species that can be intercepted by the prey species [87,88]. This interception is also called ‘eavesdropping’ [86].

Despite lacking evidence at present that Fel d 1 and mice ABP establish physical contact in nature, molecular simulations raise this possibility [14]. It would be interesting to further investigate if any interaction does happen between these proteins, in a way that could even be involved in kairomone detection. Kairomones are thought to have occurred originally as means of intraspecific communication and self-recognition in predators, outweighing any prey-alerting costs [89,90]. Rodent detection of cat kairomones would have evolved by natural selection of prey that was sensitive and avoidant to predator odor, being more likely to survive and leave offspring with similar cat-detecting traits [86].

The widespread reaction to domestic cat Fel d 1 led to research on putative orthologs in other felids. Antibody reactivity confirmed the presence of Fel d 1-like protein in lion (*Panthera leo*), leopard (*P. pardus*), jaguar *(P. onca*), tiger (*P. tigris*), snow leopard (*P. uncia*), cougar (*Puma concolor*), caracal (*Caracal caracal*), serval (*Leptailurus serval*), and ocelot (*Leopardus pardalis*) [91,92]. Nonetheless, allergy to non-domestic cats (any other member of the Felidae family than *F. catus*) seem to be extremely rare. There are only two reports on possible reactions to lion Fel d 1 [93,94], which are questionable given the environment of the cases (a zoo and a circus) and the known occurrence of cross-reactivity among furry animal allergens [95]. Considering how conspicuous are the reactions to domestic cat Fel d 1, the absence of similar reports for other felids is noteworthy, especially when one ponders that large felines are abundant in captivity, especially as “exotic pets”, outnumbering their wild counterparts [96]. In addition to that, the cases of intoxication by slow loris BGE protein are very well documented, despite being very shy nocturnal animals [4]. Besides Fel d 1, multiple felid species also share their highly specialized tongues [29].

The similarities between cat Fel d 1 and loris venom BGE protein [12,13] take part in the possible evidence for the former being considered a toxin. The BGE protein is synthesized in the brachial glands. This gland secretion is licked, becoming mixed with saliva, and filling up needle-like incisor teeth [4,11]. Humans are known to develop allergies and enter anaphylactic shock when bitten by lorises [4, 97,98]. The BGE protein is proposed to act as a communication tool among slow lorises, being able to carry different chemomessages, acting as a snare or box [98]. In this model, different molecules (from diet, saliva, and/or brachial gland) are entrapped in the BGE protein, and deposited in loris skin and fur, where they can carry messages via grooming [4]. Multiple aromatic compounds were found in the brachial gland exudate and since its earlier analysis, the presence of hydrophobic molecules was highlighted [99-102].

At the same time, lorises have protective behaviors that involve showing off the gland region in their arms when threatened, as well as biting conspecifics, causing severe tissue damage [4,11]. It has also been shown that olfaction-oriented predators avoid slow lorises, even when infants are ‘parked’ in the vegetation at the jungle floor [4]. An ectoparasite protective role has also been suggested [103]. A general comparison between BGE protein and Fel d 1 is presented in Table 1.

**Table 1.** Comparison between slow loris BGE protein and domestic cat Fel d 1.

	BGE protein	Fel d 1
Secretoglobin fold	Yes	Yes
Hydrophobic ligand binding	Yes	Yes
Glycosylation site	Probably	Yes
Allergy inducing/IgE response	Yes	Yes
Toxicity	Active/Venom	Proposed here as Passive/Poison
Interaction with saliva	Yes	Yes
Defensive role	Yes	Proposed here
Intraspecific communication	Yes	Yes
Interspecific communication	Yes	Yes
Ectoparasite resistance	Yes	Unknown

The absence of noteworthy observations of allergy against any other felid than the domestic cat, despite Fel d 1 being largely conserved, raises two main questions. One: how is Fel d 1 able to modulate human response despite being so similar to orthologs in other felids? Two: why is cat allergy still so prevalent, considering the close relationship between humans and domestic cats?

The modulation of function seems to be a staple of Fel d 1 in domestic cats. The communication role would be the primary function of this protein in all felids (independent of body size) and would be a way of intraspecific exchange along with environmental perception (by binding molecules that are present around the individual). This function is remarkably similar to the one found in the slow loris BGE protein. However, the ability to cause IgE-mediated responses (in humans, particularly) must come from additional features, considering the almost unchanged profile of Fel d 1 among felids. It has been shown that glycosylation is somewhat capable of modulating Fel d 1 conformation, and that deglycosylation alters the protein native state [21,92]. However, deglysoylated Fel d 1 was shown to induce IgE response, suggesting a lesser role for this post-translational modification in the context of cat toxicity [16,20,104]. 

The ability to bind multiple hydrophobic ligands, in the other hand, is something that not only makes Fel d 1 a perfect container to shuttle molecules between cats themselves and between cats and environment (much like what is observed for slow lorises), but also would modulate Fel d 1 toxicity. By binding different molecules, the protein is able to originate multiple conformers, thus, putatively raising multiple functions, as proposed in the protein form-function paradigm [105]. In this way, domestic cats would modulate how toxic is their Fel d 1 at any given moment by dosing different ligands (most likely endogenous and stress related). In this scenario, non-neutered male cats would require high levels of Fel d 1 to mark their territory and to monitor such territory in terms of semiochemicals, and a handling-avoidant cat would not only produce more Fel d 1, but would combine it more frequently with toxicity-causing ligands, inducing a aversive response in humans. 

The anatomical variation in Fel d 1 levels could also hint to parasite protection as a role for this protein (as proposed for lorises) [27,103]. However, no report on this function is available thus far. Since rodents eavesdrop on cat signals [86], another function of modulating Fel d 1 plasticity would be to gain some advantage in the kairomone arena.

Domestic cats still having allergy-causing phenotypes would be unexpected considering their long history of intimacy with humans. However, this is not the case. Unlike dogs, that underwent major changes due to domestication (including shifting to a starchy diet and reaching size extremes) [106,107], the so-called domestic cat (*F. catus*) is still very much unchanged regarding its ancestors [108,109]. In this sense, it is not uncommon to consider that cat domesticated themselves and that humans and cats coexist, but that no *de facto* domestication took place [110,111]. Such coexistence started in the Neolithic period in the Near East, in response to rodents targeting the surplus of grain being stored as agriculture took momentum. Wild cats are thought to have taken this opportunity to access easy prey provision in exchange of living near human groups [111]. It is plausible to think that Fel d 1 would act as a ‘human deterrent’, keeping humans at distance if necessary, considering that their presence would be secondary to feline feeding interests. Since docility seems to be the major force that shaped domestic cat genomes [109], Fel d 1 would be a countermeasure (almost as a response to being domesticated). It is also noteworthy that domestic cats underwent an expansion of their pheromone-detecting chemosensory system at the expense of odorant detection [109]. Fel d 1 most likely took on additional functions on an otherwise already in-demand communication role.

The function acquisition by Fel d 1 (and likely by BGE protein) can be considered an example of exaptation, in which features that enhance fitness were not naturally selected for their current role [112]. Considering that proteins found in animal venoms rise from a reduced set of folds, indicating functional restriction to which structures can acquire toxicity [113], it is not surprising that Fel d 1 would take on that role. It is especially interesting that its multifunctionality seem to arise from ligand variation, instead of any other protein modification. Fel d 1 and BGE protein are not only good examples of moonlighting proteins [114], but also additions to the growing list of moonlighting toxins, a group of still misidentified multifunctional proteins [115]. Such ligand-based plasticity of protein function as presented by Fel d 1 can be considered a specialized way to avoid toxin resistance, an expected outcome of interspecific toxicity coevolution [116].

The aim to reduce or eradicate cat allergy led to multiple research efforts. Allegedly hypoallergenic cats were advertised and commercialized for some time during the early 2000s [117] but are no longer available. Some cat breeds are considered hypoallergenic, but this status is not widely accepted [117,118]. Reduced levels of Fel d 1 in the fur of hypoallergenic cats have been reported [119], and at least two potentially relevant mutations were detected in Fel d 1 genes of Siberian cats, the breed most frequently listed as hypoallergenic [120]. Since such reduced levels of Fel d 1 are considered difficult to propagate [121], alternatives are currently being developed, with most of them involving some immunological intervention. Administration of monoclonal antibodies that compete with IgE for Fel d 1 were shown to reduce allergy in human patients [122]. Cat immunization against its own allergen was shown to reduce Fel d 1 levels in the animals [123], while diet supplementation with anti-Fel d 1 antibodies reduced the protein level in cat saliva [124,125]. In addition to these approaches, at least one biotechnology company is aiming to use CRISPR/Cas9 gene editing to create cats that do not synthesize Fel d 1 in their salivary glands [121]. 

As suggested by Scheib et al. [13], it is possible that researchers and personnel working with slow loris will benefit from cat-oriented treatments, considering the ample similarity between Fel d 1 and BGE protein. Cats, however, may not be unharmed by such Fel d 1-targeted approaches. Concerns, as those raised by Bienboire-Frosini et al. [81], are that, being a multifunctional protein, to eliminate it from the cat chemical repertoire would be detrimental to normal physiological and ethological functions in domestic cats. Would it be akin to neutering (widely accepted and of little consequence), to declawing (debatable but practiced), or to removing whiskers (damaging to spatial perception)? [126-128]. At this point it is not possible to state how much these treatments would affect a cat’s everyday life. 

The hypothesis presented here is based on indirect observations. *In vitro* and *in vivo* experiments on the molecular plasticity of Fel d 1 regarding its ligands (including structural determination of protein conformers, ligands, and post-translational modifications), despite being extremely complex, would most certainly answer some of the questions presented here. From a basic science point of view, this would be a unique system to be studied, which is currently under risk of being ignored once a true hypoallergenic domestic cat becomes available.

## Conclusion

Fel d 1, the major cat allergen, may satisfy some criteria to be considered a toxin. In this sense, domestic cats would be considered poisonous mammals (able to present a toxin but devoid of specialized toxin-delivery apparatus). Multiple facts seem able to support the protein toxicity as well as its role in intra- and interspecific communication. This Fel d 1 profile is strikingly similar to loris BGE protein, a secretoglobin present in slow loris venom. In both cases the variation in protein contents, instead of post-translational modifications or putative alternative splicing, act as a driving force in modulating protein activity (toxicity, in particular). This is still exploratory research (i.e. hypothesis generating), requiring further advances to move into confirmatory research (i.e. hypothesis testing). Nevertheless, the analysis of Fel d 1 from a toxinology perspective is a novelty that may aid in the understanding of this complex molecule and its effects on humans. 

## Supplementary material

The following online material is available for this article:

Additional file 1. Source information for Fel d 1 sequences used in this work. Unless otherwise noted, all accession codes pertain to NCBI.

Additional file 2. Felid and slow loris species for which protein/genomic sequence data was unavailable or was insufficient to be included in this study.

## References

[B1] Maynard CJ (1989). Singular effects produced by the bite of a short-tailed shrew, Blarina brevicauda. Cont Sci.

[B2] Dufton MJ (1992). Venomous mammals. Pharmacol Ther.

[B3] Ligabue-Braun R, Verli H, Carlini CR (2012). Venomous mammals: a review. Toxicon.

[B4] Rode-Margono JE, Nekaris KA (2015). Cabinet of curiosities: Venom systems and their ecological function in mammals, with a focus on primates. Toxins (Basel).

[B5] Ligabue-Braun R, Gopalakrishnakone P, Malhotra A (2017). Venom use in mammals: Evolutionary aspects. Evolution of Venomous Animals and Their Toxins.

[B6] Bonamonte D, Angelini G, Bonamonte D, Angelini G (2016). The aquatic biotic environment and its biotoxins. Aquatic Dermatology: Biotic, Chemical and Physical Agents.

[B7] Brodie ED (1977). Hedgehogs use toad venom in their own defence. Nature.

[B8] Kingdon J, Agwanda B, Kinnaird M, O'Brien T, Holland C, Gheysens T, Boulet-Audet M (2012). A poisonous surprise under the coat of the African crested rat. Proc Biol Sci.

[B9] Plikus MV, Astrowski AA (2014). Deadly hairs, lethal feathers - convergent evolution of poisonous integument in mammals and birds. Exp Dermatol.

[B10] Brodie III ED (2009). Toxins and venoms. Curr Biol.

[B11] Nekaris KA, Moore RS, Rode EJ, Fry BG (2013). Mad, bad and dangerous to know: the biochemistry, ecology and evolution of slow loris venom. J Venom Anim Toxins incl Trop Dis.

[B12] Krane S, Itagaki Y, Nakanishi K, Weldon PJ (2003). “Venom” of the slow loris: sequence similarity of prosimian skin gland protein and Fel d 1 cat allergen. Naturwissenschaften.

[B13] Scheib H, Nekaris KA, Rode-Margono J, Ragnarsson L, Baumann K, Dobson JS (2020). The toxicological intersection between allergen and toxin: A structural comparison of the cat dander allergenic protein Fel d1 and the slow loris brachial gland secretion protein. Toxins (Basel).

[B14] Durairaj R, Pageat P, Bienboire-Frosini C (2018). Another cat and mouse game: Deciphering the evolution of the SCGB superfamily and exploring the molecular similarity of major cat allergen Fel d 1 and mouse ABP using computational approaches. PLoS One.

[B15] Ohman JL, Lowell FC, Bloch KJ (1973). Allergens of mammalian origin: characterization of allergen extracted from cat pelts. J Allergy Clin Immunol.

[B16] Kaiser L, Grönlund H, Sandalova T, Ljunggren HG, van Hage-Hamsten M, Achour A (2003). The crystal structure of the major cat allergen Fel d 1, a member of the secretoglobin family. J Biol Chem.

[B17] Kaiser L, Velickovic TC, Badia-Martinez D, Adedoyin J, Thunberg S, Hallén D (2007). Structural characterization of the tetrameric form of the major cat allergen Fel d 1. J Mol Biol.

[B18] Klug J, Beier HM, Bernard A, Chilton BS, Fleming TP, Lehrer RI (2000). Uteroglobin/Clara cell 10-kDa family of proteins: nomenclature committee report. Ann N Y Acad Sci.

[B19] Jackson BC, Thompson DC, Wright MW, McAndrews M, Bernard A, Nebert DW (2011). Update of the human secretoglobin (SCGB) gene superfamily and an example of 'evolutionary bloom' of androgen-binding protein genes within the mouse Scgb gene superfamily. Hum Genomics.

[B20] Kristensen AK, Schou C, Roepstorff P (1997). Determination of isoforms, N-linked glycan structure and disulfide bond linkages of the major cat allergen Fel d1 by a mass spectrometric approach. Biol Chem.

[B21] Ligabue-Braun R, Sachett LG, Pol-Fachin L, Verli H (2015). The calcium goes meow: Effects of ions and glycosylation on Fel d 1, the major cat allergen. PLoS One.

[B22] Svanes C, Heinrich J, Jarvis D, Chinn S, Omenaas E, Gulsvik A (2003). Pet-keeping in childhood and adult asthma and hay fever: European community respiratory health survey. J Allergy Clin Immunol.

[B23] Bonnet B, Messaoudi K, Jacomet F, Michaud E, Fauquert JL, Caillaud D (2018). An update on molecular cat allergens: Fel d 1 and what else? Chapter 1: Fel d 1, the major cat allergen. Allergy Asthma Clin Immunol.

[B24] Grönlund H, Saarne T, Gafvelin G, van Hage M (2010). The major cat allergen, Fel d 1, in diagnosis and therapy. Int Arch Allergy Immunol.

[B25] Vervloet D, Charpin D, Birnbaum J (1995). Origine des allergènes du chat. Rev Fr Allergol.

[B26] De Andrade AD, Birnbaum J, Magalon C, Magnol JP, Lanteaume A, Charpin D (1996). Fel d I levels in cat anal glands. Clin Exp Allergy.

[B27] Carayol N, Birnbaum J, Magnan A, Ramadour M, Lanteaume A, Vervloet D (2000). Fel d 1 production in the cat skin varies according to anatomical sites. Allergy.

[B28] Kelly SM, Karsh J, Marcelo J, Boeckh D, Stepner N, Santone B (2018). Fel d 1 and Fel d 4 levels in cat fur, saliva, and urine. J Allergy Clin Immunol.

[B29] Noel AC, Hu DL (2018). Cats use hollow papillae to wick saliva into fur. Proc Natl Acad Sci USA.

[B30] Altschul SF, Gish W, Miller W, Myers EW, Lipman DJ (1990). Basic local alignment search tool. J Mol Biol.

[B31] Boratyn GM, Camacho C, Cooper PS, Coulouris G, Fong A, Ma N (2013). BLAST: a more efficient report with usability improvements. Nucleic Acids Res.

[B32] Sayers EW, Beck J, Brister JR, Bolton EE, Canese K, Comeau DC (2020). Database resources of the National Center for Biotechnology Information. Nucleic Acids Res.

[B33] Edgar RC (2004). MUSCLE: a multiple sequence alignment method with reduced time and space complexity. BMC Bioinformatics.

[B34] Larsson A (2014). AliView: a fast and lightweight alignment viewer and editor for large datasets. Bioinformatics.

[B35] Jones DT, Taylor WR, Thornton JM (1992). The rapid generation of mutation data matrices from protein sequences. Comput Appl Biosci.

[B36] Guindon S, Lethiec F, Duroux P, Gascuel O (2005). PHYML Online--a web server for fast maximum likelihood-based phylogenetic inference. Nucleic Acids Res.

[B37] Anisimova M, Gascuel O (2006). Approximate likelihood-ratio test for branches: A fast, accurate, and powerful alternative. Syst Biol.

[B38] Lefort V, Longueville JE, Gascuel O (2017). SMS: Smart model selection in PhyML. Mol Biol Evol.

[B39] Pettersen EF, Goddard TD, Huang CC, Couch GS, Greenblatt DM, Meng EC (2004). UCSF Chimera--a visualization system for exploratory research and analysis. J Comput Chem.

[B40] Johnson WE, Eizirik E, Pecon-Slattery J, Murphy WJ, Antunes A, Teeling E (2006). The late Miocene radiation of modern Felidae: a genetic assessment. Science.

[B41] Li G, Figueiró HV, Eizirik E, Murphy WJ (2019). Recombination-aware phylogenomics reveals the structured genomic landscape of hybridizing cat species. Mol Biol Evol.

[B42] Gould HJ, Sutton BJ (2008). IgE in allergy and asthma today. Nat Rev Immunol.

[B43] Stanworth DR (1987). The "discovery" of IgE. Allergol Immunopathol (Madr).

[B44] Stanworth DR (1993). The discovery of IgE. Allergy.

[B45] Galli SJ, Tsai M (2012). IgE and mast cells in allergic disease. Nat Med.

[B46] Kelly BT, Grayson MH (2016). Immunoglobulin E, what is it good for?. Ann Allergy Asthma Immunol.

[B47] Profet M (1991). The function of allergy: immunological defense against toxins. Q Rev Biol.

[B48] Palm NW, Rosenstein RK, Medzhitov R (2012). Allergic host defences. Nature.

[B49] Mukai K, Tsai M, Starkl P, Marichal T, Galli SJ (2016). IgE and mast cells in host defense against parasites and venoms. Semin Immunopathol.

[B50] Tsai M, Starkl P, Marichal T, Galli SJ (2015). Testing the ‘toxin hypothesis of allergy’: mast cells, IgE, and innate and acquired immune responses to venoms. Curr Opin Immunol.

[B51] Galli SJ, Starkl P, Marichal T, Tsai M (2016). Mast cells and IgE in defense against venoms: Possible “good side” of allergy?. Allergol Int.

[B52] Mendes E, Ulhôa Cintra A, Corrêa A (1960). Allergy to snake venoms. J Allergy.

[B53] Reimers AR, Weber M, Müller UR (2000). Are anaphylactic reactions to snake bites immunoglobulin E-mediated?. Clin Exp Allergy.

[B54] Priyankara WDD, Manoj EM, Gunapala A, Ranaweera AGRMA, Vithanage KS, Sivasubramanium M (2019). Cardiogenic Shock due to Kounis Syndrome following Cobra Bite. Case Rep Crit Care.

[B55] Zink A, Schuster B, Winkler J, Eyerich K, Darsow U, Brockow K (2019). Allergy and sensitization to Hymenoptera venoms in unreferred adults with a high risk of sting exposure. World Allergy Organ. J.

[B56] Mohd Adnan K (2018). A review on respiratory allergy caused by insects. Bioinformation.

[B57] Prescott RA, Potter PC (2005). Hypersensitivity to airborne spitting cobra snake venom. Ann Allergy Asthma Immunol.

[B58] Koh SH, Ponampalam R, Lim SH, Gunasekeran DV (2015). Cutaneous exposure to cobra venom: an uncommon presentation. Wilderness Environ Med.

[B59] de Medeiros CR, Barbaro KC, Lira MS, França FOS, Zaher VL, Kokron CM (2008). Predictors of Bothrops jararaca venom allergy in snake handlers and snake venom handlers. Toxicon.

[B60] Madero MF, Gámez C, Madero MA, Fernández-Nieto M, Sastre J, del Pozo V (2009). Characterization of allergens in four South American snake species. Int Arch Allergy Immunol.

[B61] de Pontes LG, Cavassan NR, Creste CF, Lourenço A, Arcuri HA, Ferreira RS (2016). Crotoxin: a novel allergen to occupational anaphylaxis. Ann Allergy Asthma Immunol.

[B62] Pontes-de-Carvalho L, Mengel J (2014). A question of nature: Some antigens are bound to be allergens. Front Immunol.

[B63] Laukaitis CM, Karn RC (2005). Evolution of the secretoglobins: a genomic and proteomic view. Biol J Linn Soc.

[B64] West LC, Grotzke JE, Cresswell P (2013). MHC class II-restricted presentation of the major house dust mite allergen Der p 1 Is GILT-dependent: implications for allergic asthma. PLoS One.

[B65] Craik DJ, Daly NL, Waine C (2001). The cystine knot motif in toxins and implications for drug design. Toxicon.

[B66] Govindu PCV, Chakraborty P, Dutta A, Gowd KH (2017). Structural space of intramolecular peptide disulfides: Analysis of peptide toxins retrieved from venomous peptide databases. Comput Biol Chem.

[B67] Bateman BJ, Dean TP (1999). The Cheshire cat’s grin - is cat allergy here to stay?. Clin Exp Allergy.

[B68] Munir AKM, Einarsson R, Schou C, Dreborg SKG (1993). Allergens in school dust. I. The amount of major cat (Fel d 1) and dog (Can f 1) allergen in dust from Swedish schools is high enough to probably cause perennial symptoms in most children with asthma who are sensitised to cat and dog. J Allergy Clin Immunol.

[B69] Custovic A, Taggart SCO, Woodcock A (1994). House dust mite and cat allergen in different indoor environments. Clin Exp Allergy.

[B70] Munir AK, Einarsson R Dreborg SK (1995). Mite (Der p I, Der f I), cat (Fel d I) and dog (Can f I) allergens in dust from Swedish day-care centres. Clin Exp Allergy.

[B71] D’Amato G, Liccardi G, Russo M, Barber D, D’Amato M, Carreira J (1997). Clothing is a carrier of cat allergens. J Allergy Clin Immunol.

[B72] Justino CM, Segundo GR, Pereira FL, Silva DA, Sopelete MC, Sung SS (2005). Mite and pet allergen exposure in Brazilian private cars. Ann Allergy Asthma Immunol.

[B73] Niesler A, Ścigała G, Łudzeń-Izbińska B (2016). Cat (Fel d 1) and dog (Can f 1) allergen levels in cars, dwellings and schools. Aerobiologia.

[B74] Chan-Yeung M, McClean PA, Sandell PR, Slutsky AS, Zamel N (1999). Sensitization to cat without direct exposure to cats. Clin Exp Allergy.

[B75] Wood RA Laheri, AN Eggleston PA (1993). The aerodynamic characteristics of cat allergen. Clin Exp Allergy.

[B76] Custovic A, Simpson A, Pahdi H, Green RM, Chapman MD, Woodcock A (1998). Distribution, aerodynamic characteristics, and removal of the major cat allergen Fel d 1 in British homes. Thorax.

[B77] Wood RA, Chapman MD, Adkinson NF, Eggleston PA (1989). The effect of cat removal on allergen content in household dust samples. J Allergy Clin Immunol.

[B78] De Blay F, Chapman MD, Platts-Mills TAE (1990). Airborne cat allergens: Factors influencing the concentration of airborne cat allergen (Fel d 1). Clin Exp Allergy.

[B79] Avner DB, Perzanowski MS, Platts-Mills TA, Woodfolk JA (1997). Evaluation of different techniques for washing cats: Quantitation of allergen removed from the cat and the effect on airborne Fel d 1. J Allergy Clin Immunol.

[B80] Bienboire-Frosini C, Cozzi A, Lafont-Lecuelle C, Vervloet D, Ronin C, Pageat P (2012). Immunological differences in the global release of the major cat allergen Fel d 1 are influenced by sex and behaviour. Vet J.

[B81] Bienboire-Frosini C, Durairaj R, Pelosi P, Pageat P (2020). The major cat allergen Fel d 1 binds steroid and fatty acid semiochemicals: A combined in silico and in vitro study. Int J Mol Sci.

[B82] Mukherjee AB, Zhang Z, Chilton BS (2007). Uteroglobin: a steroid-inducible immunomodulatory protein that founded the Secretoglobin superfamily. Endocr Rev.

[B83] Karn RC (1994). The mouse salivary androgen-binding protein (ABP) alpha subunit closely resembles chain 1 of the cat allergen Fel d I. Biochem Genet.

[B84] Pageat P, Gaultier E (2003). Current research in canine and feline pheromones. Vet Clin North Am Small Anim Pract.

[B85] Staples LG, Hunt GE, van Nieuwenhuijzen PS, McGregor IS (2008). Rats discriminate individual cats by their odor: possible involvement of the accessory olfactory system. Neurosci Biobehav Rev.

[B86] May MD, Bowen MT, McGregor IS, Timberlake W (2012). Rubbings deposited by cats elicit defensive behavior in rats. Physiol Behav.

[B87] Brown WL, Eisner T, Whittaker RH (1970). Allomones and kairomones: Transpecific chemical messengers. Bio Sci.

[B88] Pasteels JM (1982). Is kairomone a valid and useful term?. J Chem Ecol.

[B89] Sbarbati A, Osculati F (2006). Allelochemical communication in vertebrates: kairomones, allomones and synomones. Cells Tissues Organs.

[B90] Papes F, Logan DW, Stowers L (2010). The vomeronasal organ mediates interspecies defensive behaviors through detection of protein pheromone homologs. Cell.

[B91] de Groot H, van Swieten P, Aalberse RC (1990). Evidence for a Fel d I-like molecule in the "big cats" (Felidae species). J Allergy Clin Immunol.

[B92] Vailes LD, Li Y, Bao Y, de Groot H, Aalberse RC, Chapman MD (1994). Fine specificity of B-cell epitopes on Felis domesticus allergen I (Fel d I): effect of reduction and alkylation or deglycosylation on Fel d I structure and antibody binding. J Allergy Clin Immunol.

[B93] Blamoutier P (1963). Quelques curieux cas d'allergie a divers poils d'animaux. Rev Franç d'Allergy.

[B94] Feleszko W, Zalewski BM, Kulus M (2014). Unexpected cross-reactivity in a cat-allergy patient. An allergic reaction at the circus. Allergol Immunopathol (Madr).

[B95] Liccardi G, Salzillo A, Steinhilber G, Meriggi A, Piccolo A, D'Amato G (2014). Is generalized reaction after exposure to big cats at the circus really unpredictable in highly cat-allergic individuals?. Allergol Immunopathol.

[B96] Nayhus PJ, Tilson R, Hutchins M, Tilson R, Nayhus PJ (2010). Thirteen Thousand and Counting: How Growing Captive Tiger Populations Threaten Wild Tigers. Tigers of The World: The Science, Politics, and Conservation of *Panthera tigris*.

[B97] Wilde H (1972). Anaphylactic shock following bite by a ‘Slow loris’, Nycticebus coucang. Am J Trop Med Hyg.

[B98] Madani G, Nekaris KAI (2014). Anaphylactic shock following the bite of a wild Kayan slow loris (Nycticebus kayan): implications for slow loris conservation. J Venom Anim Toxins incl Trop Dis.

[B99] Hagey LR, Fry BG, Fitch-Snyder H, Gursky SL, Nekaris KAI (2007). Talking defensively, a dual use for the brachial gland exudates of slow and pygmy lorises. Primate anti-predator strategies.

[B100] Alterman L (1989). Analysis of organic extracts of brachial gland exudate from Nycticebus coucang. Am J Primatol.

[B101] Alterman L (1990). Isolation of toxins from brachial gland exudates from Nycticebus coucang. Am J Phys Anthropol.

[B102] Alterman L, Hale ME (1991). Comparisons of toxins from brachial gland exudates of Nycticebus coucang and N. pygmaeus. Am J Phys Anthropol.

[B103] Grow NB, Wirdateti -, Nekaris KA (2015). Does toxic defence in Nycticebus spp. relate to ectoparasites? The lethal effects of slow loris venom on arthropods. Toxicon.

[B104] Seppälä U, Hägglund P, Wurtzen PA, Ipsen H, Thorsted P, Lenhard T (2005). Molecular characterization of major cat allergen Fel d 1: expression of heterodimer by use of a baculovirus expression system. J Biol Chem.

[B105] Richardson JS (1981). The anatomy and taxonomy of protein structure. Adv Protein Chem.

[B106] Axelsson E, Ratnakumar A, Arendt ML, Maqbool K, Webster MT, Perloski M (2013). The genomic signature of dog domestication reveals adaptation to a starch-rich diet. Nature.

[B107] Freedman AH, Wayne RK (2017). Deciphering the origin of dogs: From fossils to genomes. Annu Rev Anim Biosci.

[B108] Driscoll CA, Menotti-Raymond M, Roca AL, Hupe K, Johnson WE, Geffen E (2007). The Near Eastern origin of cat domestication. Science.

[B109] Montague MJ, Li G, Gandolfi B, Khan R, Aken BL, Searle SM (2014). Comparative analysis of the domestic cat genome reveals genetic signatures underlying feline biology and domestication. Proc Natl Acad Sci USA.

[B110] Driscoll CA, Clutton-Brock J, Kitchener AC, O’Brien SJ (2009). The taming of the cat. Sci Am.

[B111] Ottoni C, Van Neer W, De Cupere B (2017). The palaeogenetics of cat dispersal in the ancient world. Nat Ecol Evol.

[B112] Gould SJ, Vrba ES (1982). Exaptation - a missing term in the science of form. Paleobiology.

[B113] Fry BG, Roelants K, Champagne DE, Scheib H, Tyndall JD, King GF (2009). The toxicogenomic multiverse: convergent recruitment of proteins into animal venoms. Annu Rev Genomics Hum Genet.

[B114] Jeffery CJ (2018). Protein moonlighting: what is it, and why is it important?. Philos Trans R Soc Lond B Biol Sci.

[B115] Ligabue-Braun R, Carlini CR, Gopalakrishnakone P, Carlini CR, Ligabue-Braun R (2017). Moonlighting toxins: Ureases and beyond. Plant Toxins.

[B116] Arbuckle K, Rodríguez de la Vega RC, Casewell NR (2017). Coevolution takes the sting out of it: Evolutionary biology and mechanisms of toxin resistance in animals. Toxicon.

[B117] Butt A, Rashid D, Lockey RF (2012). Do hypoallergenic cats and dogs exist?. Ann Allergy Asthma Immunol.

[B118] Chan SK, Leung DYM (2018). Dog and cat allergies: Current state of diagnostic approaches and challenges. Allergy Asthma Immunol Res.

[B119] Satorina J, Szalai K, Willensdorfer A, Mothes-Luksch N, Lukschal A, Jensen-Jarolim E (2014). Do hypoallergenic cats exist? - Determination of major cat allergen Fel d 1 production in normal and hypoallergenic cat breeds. Clin Transl Allergy.

[B120] Sartore S, Landoni E, Maione S, Tarducci A, Borrelli A, Soglia D (2017). Polymorphism analysis of Ch1 and Ch2 genes in the Siberian cat. Vet Sci.

[B121] Engelhaupt E (2020). With a litter of tactics, scientists work to tame cat allergies. ScienceNews.

[B122] Orengo JM, Radin AR, Kamat V, Badithe A, Ben LH, Bennett BL (2018). Treating cat allergy with monoclonal IgG antibodies that bind allergen and prevent IgE engagement. Nat Commun.

[B123] Thoms F, Jennings GT, Maudrich M, Vogel M, Haas S, Zeltins A (2019). Immunization of cats to induce neutralizing antibodies against Fel d 1, the major feline allergen in human subjects. J Allergy Clin Immunol.

[B124] Satyaraj E, Gardner C, Filipi I, Cramer K, Sherrill S (2019). Reduction of active Fel d1 from cats using an antiFel d1 egg IgY antibody. Immun Inflamm Dis.

[B125] Satyaraj E, Wedner HJ, Bousquet J (2019). Keep the cat, change the care pathway: A transformational approach to managing Fel d 1, the major cat allergen. Allergy.

[B126] Rauschecker JP (1995). Compensatory plasticity and sensory substitution in the cerebral cortex. Trends Neurosci.

[B127] Patronek GJ (2001). Assessment of claims of short- and long-term complications associated with onychectomy in cats. J Am Vet Med Assoc.

[B128] Welsh P (2018). Cat neutering: the earlier the better to tackle overpopulation. Vet Rec.

