# Supplementary material for: Hello, kitty: could cat allergy be a form of intoxication?
Source: J Venom Anim Toxins Incl Trop Dis. 2020 Dec 14;26:e20200051. doi: 10.1590/1678-9199-JVATITD-2020-0051 (PMC7781471; doi:10.1590/1678-9199-JVATITD-2020-0051)
Supplement: Additional file 1. [file 1678-9199-jvatitd-26-e20200051-s1.pdf]

J Venom Anim Toxins incl Trop Dis,
https://doi.org/10.1590/1678-9199-JVATITD-2020-0051

Supplementary Material to "Hello, kitty: could cat allergy be a form of intoxication?"

Additional file 1. Source information for Fel d 1 sequences used in this work. Unless otherwise noted, all accession codes pertain to NCBI.

Species	Common name	Accession [Chain 1]	Accession [Chain 2]
Acinonyx jubatus	cheetah	XP_014928582.1	XP_014928584.1
Catopuma badia	bay cat	SRX3213495	SRX3213495
Catopuma temminckii	Asiatic golden cat	SRX3213486	SRX3213486
Felis catus	domestic cat	UniProtKB - P30438	UniProtKB - P30440
Felis chaus	jungle cat	SRX1058146	SRX1058146
Felis margarita	sand cat	SRX1058385	SRX1058385
Felis nigripes	black-footed cat	SRX1284339	SRX1284339
Felis silvestris	European wildcat	SRX026960	SRX026960
Herpailurus yagouaroundi	jaguarundi	SRX3213492	SRX3213492
Leopardus colocolo	Pampas cat	SRX3213485	SRX3213485
Leopardus geoffroyi	Geoffroy's cat	SRX3213482	SRX3213482
Leopardus pardalis	ocelot	SRX3213484	SRX3213484
Leopardus tigrinus	little spotted cat	SRX3213483	SRX3213483
Leptailurus serval	serval	SRX3213491	SRX3213491
Lynx canadensis	Canada lynx	VHLF01007608.1	VHLF01007608.1
Lynx pardinus	Iberian lynx	CAAGRJ010036227.1	CAAGRJ010036227.1
Lynx rufus	bobcat	SRX3213494	SRX3213494
Neofelis nebulosa	clouded leopard	SRX7041772	SRX7041772
Otocolobus manul	Palla's cat	SRX2366372	SRX2366372
Panthera leo	lion	VOCV01000308.1	VOCV01000308.1
Panthera onca	jaguar	PISV01016950.1	PISV01016950.1
Panthera pardus	leopard	XP_019278942.1	XP_019278941.1
Panthera tigris	tiger	XP_007096805.1	XP_007096807.1
Pardofelis marmorata	marbled cat	SRX3213490	SRX3213490
Prionailurus bengalensis	leopard cat	BIMV01009660.1	BIMV01009660.1
Prionailurus planiceps	flat-headed cat	SRX3213488	SRX3213488
Prionailurus rubiginosus	rusty-spotted cat	SRX3213487	SRX3213487
Prionailurus viverrinus	fishing cat	SRX3213489	SRX3213489
Puma concolor	cougar	PSOM01131472.1	PSOM01131472.1

Nycticebus coucang	slow loris	PVIV000000000.1	PVIV000000000.1
Nycticebus javanicus	Javan slow loris	From Scheib et al. [13]	From Scheib et al. [13]
Nycticebus pygmaeus	pygmy slow loris	SRX5112421	SRX5112421
