# Supplementary material for: Hello, kitty: could cat allergy be a form of intoxication?
Source: J Venom Anim Toxins Incl Trop Dis. 2020 Dec 14;26:e20200051. doi: 10.1590/1678-9199-JVATITD-2020-0051 (PMC7781471; doi:10.1590/1678-9199-JVATITD-2020-0051)
Supplement: Additional file 2. [file 1678-9199-jvatitd-26-e20200051-s2.pdf]

J Venom Anim Toxins incl Trop Dis,
https://doi.org/10.1590/1678-9199-JVATITD-2020-0051

Supplementary Material to "Hello, kitty: could cat allergy be a form of intoxication?"

Additional file 2. Felid and slow loris species for which protein/genomic sequence data was unavailable or was insufficient to be included in this study.

Species	Common name
Caracal aurata	African golden cat
Caracal caracal	caracal
Felis bieti	Chinese mountain cat
Felis lybica	African wildcat
Leopardus guigna	kodkod
Leopardus guttulus	Southern tiger cat
Leopardus jacobita	Andean mountain cat
Leopardus wiedii	margay
Lynx lynx	Eurasian lynx
Neofelis diardi	Sunda clouded leopard
Panthera uncia	snow leopard
Prionailurus javanensis	Sunda leopard cat

Nycticebus bengalensis	Bengal slow loris
Nycticebus kayan	Kayan River slow loris
Nycticebus menagensis	Bornean slow loris
